# Simultaneous Pancreas-Kidney Transplantation Versus Kidney Transplantation Alone in Type 1 Diabetes: Does Pancreas Transplantation Improve Clinical Outcomes? A Systematic Review and Exploratory Meta-Analysis

**DOI:** 10.3390/medsci14040454

**Published:** 2026-08-03

**Authors:** Maria Irene Bellini, Claudia De Intinis, Gabriele D’Andrea, Vito D’Andrea, Maurizio Vichi

**Affiliations:** 1 Department of Surgery, Sapienza University, 00161 Rome, Italy; 2 Department of Statistical Sciences, Sapienza University, 00161 Rome, Italy

**Keywords:** type 1 diabetes mellitus (T1DM), end-stage renal disease (ESRD), simultaneous pancreas–kidney transplantation (SPKT), living-donor kidney transplantation (LDKT), deceased-donor kidney transplantation (DDKT), patient survival, kidney graft survival, systematic review and meta-analysis

## Abstract

Background: Simultaneous pancreas–kidney transplantation (SPKT) restores both renal function and endogenous insulin secretion in selected patients with type 1 diabetes mellitus (T1DM) and end-stage renal disease (ESRD). Whether SPKT provides superior patient survival, kidney graft outcomes and cardiovascular benefit compared with kidney transplantation alone (KTA) remains debated, particularly when KTA is performed from a living donor. Methods: A systematic review was conducted according to PRISMA 2020 guidelines. PubMed/MEDLINE was searched using a predefined strategy including terms related to pancreas transplantation, kidney transplantation alone, T1DM, and ESRD/chronic kidney disease. Eligible studies included adult T1DM/ESRD populations comparing SPKT with KTA, including living-donor kidney transplantation (LDKT) and deceased-donor kidney transplantation (DDKT) and reporting clinically relevant outcomes. Full texts were reviewed and categorized as core comparative evidence, secondary/supportive evidence or excluded records. A quantitative synthesis was additionally performed for studies reporting directly comparable adjusted hazard ratios for patient mortality and kidney graft failure in the SPKT versus LDKT comparison. Results: Nineteen observational studies met the inclusion criteria and were included in the qualitative synthesis. SPKT consistently provided superior metabolic control and insulin independence when pancreas graft function was maintained. Compared with deceased-donor or mixed KTA cohorts, SPKT was frequently associated with more favorable long-term patient survival and cardiovascular outcomes in selected recipients. However, comparisons with LDKT yielded less consistent results, with several registry-based analyses reporting equivalent or superior kidney graft and survival outcomes after living-donor transplantation. Quantitative synthesis of the two studies providing directly comparable adjusted hazard ratios demonstrated a higher risk of patient mortality (HR 1.30, 95% CI 1.10–1.54) and kidney graft failure (HR 1.43, 95% CI 1.24–1.66) following SPKT compared with LDKT. Formal meta-analysis of SPKT versus DDKT was not feasible because of substantial heterogeneity in outcome definitions, statistical reporting methods and follow-up duration across studies. Conclusions: In adults with T1DM and ESRD, successful SPKT provides a durable metabolic advantage and may improve long-term outcomes compared with deceased-donor KTA in selected patients. Across analyses that included all transplanted recipients from the time of surgery (intent-to-treat), early perioperative risk is higher after SPKT but may be offset over time when pancreas graft function is maintained. Evidence does not support a universal survival superiority of SPKT over living-donor kidney transplantation. Our quantitative synthesis of intent-to-treat, transplant-date analyses indicates that LDKT is associated with lower risks of patient mortality and kidney graft failure compared with SPKT when a suitable living donor is available. Treatment decisions should be individualized, considering living-donor availability, anticipated waiting time and dialysis exposure, cardiovascular and surgical risk, and the likelihood of durable pancreas graft function, with greater weight given to contemporary cohorts.

## 1. Introduction

Type 1 diabetes mellitus remains a major cause of end-stage renal disease [1,2]. Kidney transplantation improves survival and quality of life compared with dialysis, but kidney transplantation alone does not correct the underlying diabetic state [3]. Simultaneous pancreas-kidney transplantation offers both renal replacement and restoration of endogenous insulin secretion, with the potential for insulin independence, improved glycemic control and stabilization or improvement of diabetic complications [4,5,6].

The central clinical controversy is not whether SPKT can normalize glucose metabolism, but whether the additional pancreas transplant translates into better patient survival, kidney graft survival and cardiovascular outcomes when compared with KTA. This question is particularly complex because KTA includes biologically and clinically distinct comparators: living-donor KTA, deceased-donor KTA and mixed KTA cohorts. In addition, SPKT recipients are often younger and more stringently selected, while KTA recipients may have different cardiovascular risk, dialysis exposure and donor characteristics.

This systematic review evaluates comparative clinical outcomes of SPKT versus KTA in adult patients with T1DM and ESRD, with emphasis on patient survival, kidney graft outcomes, cardiovascular/metabolic outcomes and procedure-related trade-offs.

## 2. Methods

### 2.1. Review Question and Design

This review aimed to evaluate the comparative effectiveness of simultaneous pancreas-kidney transplantation and kidney transplantation alone in adult patients with type 1 diabetes mellitus and end-stage renal disease or advanced chronic kidney disease (CKD), with particular focus on patient survival, kidney graft survival and function, cardiovascular outcomes, metabolic outcomes, complications and quality of life. The review was designed as a systematic review with narrative synthesis, complemented by an exploratory quantitative meta-analysis of eligible studies reporting directly comparable adjusted hazard ratios. Eligibility criteria were predefined according to the PICO framework and are summarized in Table 1. PROSPERO registration number is CRD420261445643.

### 2.2. Search Strategy and Study Selection

A predefined PubMed/MEDLINE search string was applied: (“Pancreas Transplantation”[Mesh] OR “simultaneous pancreas kidney transplantation”[tiab] OR “simultaneous pancreas-kidney transplantation”[tiab] OR “simultaneous kidney pancreas transplantation”[tiab] OR SPKT[tiab] OR SKP[tiab]) AND (“Kidney Transplantation”[Mesh] OR “kidney transplant alone”[tiab] OR “kidney transplantation alone”[tiab] OR KTA[tiab]) AND (“Diabetes Mellitus, Type 1”[Mesh] OR “type 1 diabetes”[tiab] OR T1DM[tiab] OR IDDM[tiab]) AND (“Kidney Failure, Chronic”[Mesh] OR ESRD[tiab] OR “end stage renal disease”[tiab] OR CKD[tiab] OR “chronic kidney disease”[tiab] OR “Diabetic Nephropathies”[Mesh] OR “diabetic nephropathy”[tiab]).

The PubMed/MEDLINE search was last performed in June 2026, with no lower date limit (database inception to June 2026). Automated filters retained English-language human adult studies and excluded preprints. Eligible article types included clinical, comparative, observational, multicenter and evaluation studies. Titles and abstracts were screened in Rayyan^®^, Cambridge, MA 02142 USA, followed by full-text assessment of potentially eligible reports [7]. Data were assessed independently by two reviewers, CDI and MIB. Conflicts were solved by consulting a third author, VDA. When multiple publications reported overlapping cohorts, the publication with the longest follow-up and most complete outcome data was used for the primary synthesis, while earlier reports were retained only for contextual information.

### 2.3. Data Extraction

For each included study, extracted variables included: author, year, country, study design, data source, population, intervention, comparator, sample size, follow-up duration, outcomes and numerical results. Data were extracted into a master evidence table and cross-checked against the original full-text articles.

### 2.4. Risk of Bias Assessment and Synthesis Approach

The methodological quality of the included observational studies was assessed using the Newcastle–Ottawa Scale (NOS) for cohort studies, which evaluates three domains: selection of cohorts, comparability of study groups, and outcome assessment including adequacy of follow-up [8].

For the selection domain, studies were judged according to the representativeness of the SPKT and KTA cohorts, the definition of transplant exposure and the inclusion of clinically relevant type 1 diabetic patients with ESRD or advanced CKD. For the comparability domain, particular emphasis was placed on whether analyses accounted for major confounders, including recipient age, donor source, living versus deceased donor transplantation, dialysis exposure, cardiovascular comorbidity, transplant era and baseline differences between SPKT and KTA recipients. For the outcome domain, studies were evaluated according to the objectivity of outcome assessment, duration of follow-up and completeness of outcome reporting.

A broad meta-analysis across all included studies was not performed because substantial clinical and methodological heterogeneity was identified across the evidence base. Comparator groups varied considerably (living-donor kidney transplantation, deceased-donor kidney transplantation and mixed kidney transplantation-alone cohorts), follow-up duration ranged from 6 months to 10 years, outcome definitions were not uniform and reported effect measures included survival proportions, hazard ratios, restricted mean survival time, renal function parameters and event rates. Findings were therefore mainly synthesized using a structured narrative approach.

### 2.5. Quantitative Synthesis and Meta-Analysis

A quantitative synthesis was performed for studies reporting adjusted hazard ratios (HRs) and corresponding 95% confidence intervals (CIs) for direct comparisons between simultaneous pancreas–kidney transplantation (SPKT) and living-donor kidney transplantation (LDKT). Only studies reporting directly comparable adjusted hazard ratios and corresponding confidence intervals for both patient mortality and kidney graft failure were considered eligible for quantitative synthesis. Given the heterogeneity of outcome definitions, statistical reporting methods and follow-up intervals across the remaining studies, quantitative pooling was restricted to patient mortality and kidney graft failure outcomes in the SPKT versus LDKT comparison.

To ensure a uniform interpretation across studies, all effect estimates were standardized to the direction SPKT versus LDKT. For studies reporting outcomes as LDKT versus SPKT, hazard ratios were transformed using reciprocal conversion (1/HR). Corresponding 95% confidence intervals were transformed by taking the reciprocal of the upper and lower confidence limits, respectively. Consequently, HR values greater than 1.0 indicate a higher risk associated with SPKT, whereas HR values lower than 1.0 favor SPKT over LDKT.

Log-transformed hazard ratios and standard errors derived from the reported confidence intervals were pooled using the inverse-variance fixed-effect model. Statistical heterogeneity was assessed using Cochran’s Q statistic and the I^2^ statistic.

## 3. Results

### 3.1. Study Selection and Characteristics of the Evidence Base

**Figure 1:**
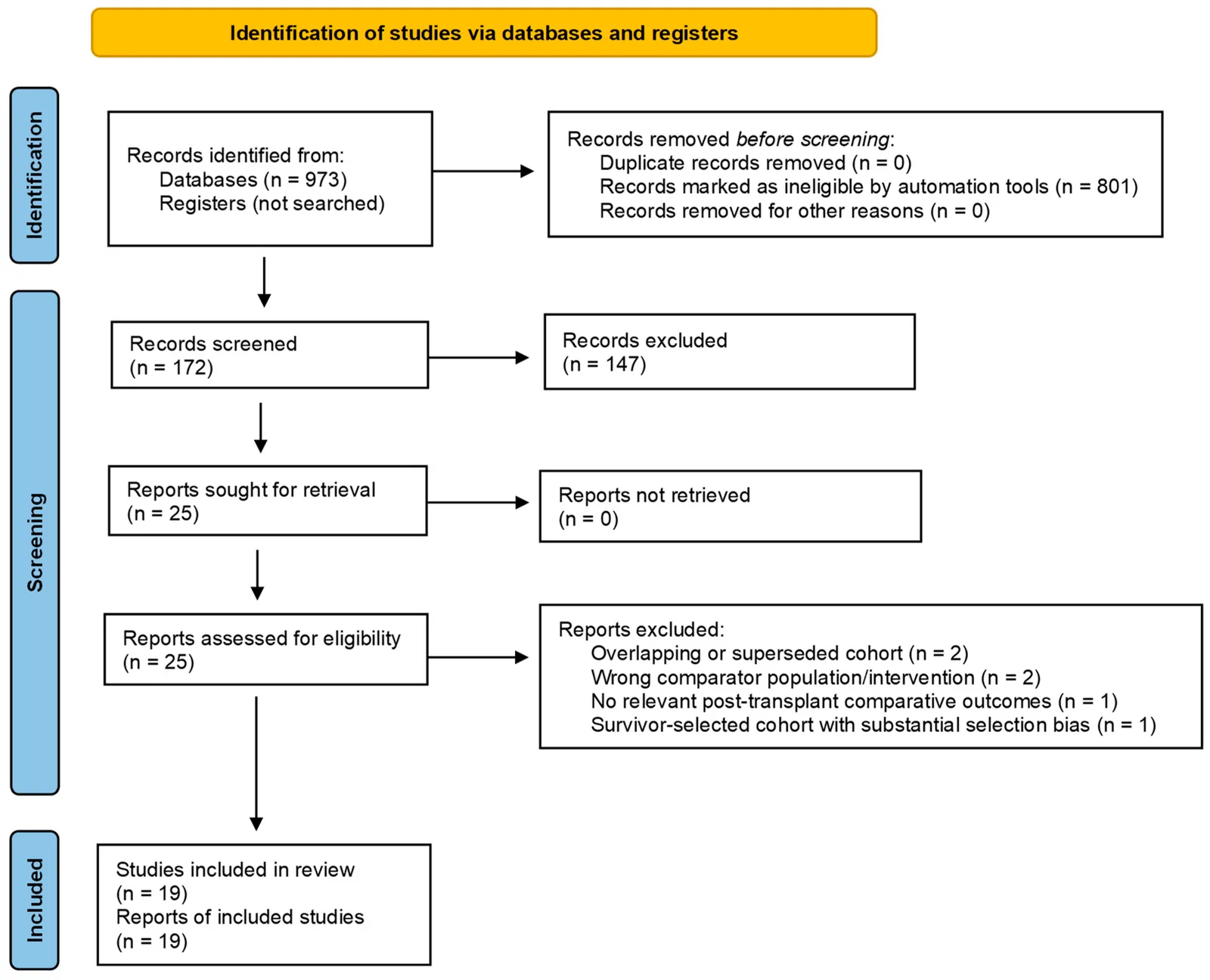
PRISMA 2020 flow diagram of study selection.

The literature search and study selection process are summarized in the PRISMA 2020 flow diagram (Figure 1) [9].

The literature search yielded 973 records, of which 172 remained after application of predefined database filters. Following title and abstract screening, 25 articles were selected for full-text assessment.

After full-text evaluation, 19 studies were retained for qualitative synthesis. These comprised 16 core comparative studies that directly addressed the review question and 3 secondary/supportive studies that provided contextual evidence regarding long-term outcomes, metabolic effects, cardiovascular complications, quality-of-life considerations or methodological interpretation. Six reports were not retained in the final evidence synthesis because they represented duplicate or overlapping cohorts, earlier versions of subsequently updated datasets, inappropriate comparators, review articles or studies falling outside the predefined review scope. When overlapping cohorts were identified, only the publication with the longest follow-up and most complete outcome data was retained for the primary synthesis.

The included evidence consisted exclusively of observational comparative studies published between 1995 and 2025, ranging from single-centre retrospective cohorts to large national registry analyses. Detailed characteristics of the 16 core comparative studies are presented in Table 2 [10,11,12,13,14,15,16,17,18,19,20,21,22,23,24,25] and Supplementary Table S1 [10,11,12,13,14,15,16,17,18,19,20,21,22,23,24,25]. Table 3 [10,11,12,13,14,15,16,17,18,19,20,21,22,23,24,25] reports NOS assessment. The contribution of secondary/supportive studies to the interpretation of the evidence base is summarized in Supplementary Table S2 [21,26,27], while studies assessed at full-text level but not retained in the final synthesis are reported in Supplementary Table S3 [28,29,30,31,32,33].

### 3.2. Intent-to-Treat Reporting and Inclusion of Early Mortality

In most registry-based comparative studies (Reddy 2003 [16]; Young 2009 [18]; Sung 2015 [19]; Barlow 2017 [21]; Catarinella 2025 [24]; Budhiraja 2025 [25]), outcomes were analyzed from the date of transplant and thus included early post-operative deaths (intent-to-treat at transplant). In contrast, a minority of studies employed survivor-enriched or as-treated approaches; Biesenbach 2005 [22], for example, analyzed only recipients with functioning grafts beyond 5 years (page 8, lines 227–229), and one survivor-selected report was excluded at full-text due to substantial selection bias (Figure 1, page 5, lines 157–162). Where applicable, we reference these design features in the narrative synthesis and Risk-of-Bias assessment.

### 3.3. Patient Survival

The survival evidence was comparator-dependent. Historical data from Manske et al. suggested substantially higher early mortality after simultaneous pancreas–kidney transplantation (SPKT) compared with cadaveric KTA, largely driven by infectious complications and pancreatectomy [10].

Later single-centre studies and registry analyses more frequently reported favorable survival outcomes after SPKT compared with deceased-donor or mixed KTA cohorts, particularly when pancreas graft function was maintained. Mohan et al. reported superior survival after SPKT at 8 years [15]. Reddy et al. [16] found that SPKT and living-donor kidney transplant recipients had similar 8-year survival, both outperforming cadaveric kidney transplant recipients, while also demonstrating an early mortality disadvantage for SPKT during the first 18 months after transplantation [16]. Sung et al. reported a statistically significant but clinically modest adjusted restricted mean survival advantage for SPKT compared with KTA over 10 years [19]. Lange et al. similarly reported improved survival after SPKT in a propensity-matched single-centre cohort [23].

More recent, rigorously adjusted registry evidence is more nuanced. In a large global propensity score–matched cohort, Catarinella et al. found that the crude survival advantage of SPKT over KTA did not persist after matching (all-cause mortality HR 1.00, 95% CI 0.90–1.10), whereas SPKT recipients maintained significantly lower HbA1c levels throughout follow-up [24]. Likewise, after overlap propensity-score weighting, Budhiraja et al. found no significant patient-survival advantage of SPKT over deceased-donor kidney transplantation, and reported greater early morbidity in the SPKT group [25].

In contrast, comparisons with living-donor KTA yielded less favorable results for SPKT. Young et al. reported lower adjusted risks of patient death and kidney graft failure after LDKT compared with SPKT [18]. Barlow et al. found no overall patient survival advantage of SPKT over LDKT in the UK registry, although recipients with a functioning pancreas graft experienced better outcomes than those with pancreas graft failure [21]. Overall, the available evidence suggests a potential survival advantage of successful SPKT over deceased-donor or mixed KTA in selected recipients, but not a consistent superiority over living-donor KTA.

### 3.4. Kidney Graft Survival and Kidney Function

Kidney graft outcomes were broadly consistent with the survival findings. SPKT generally compared favorably with deceased-donor KTA in several cohorts, whereas living-donor KTA remained a highly competitive alternative. Douzdjian et al. reported similar 1- and 5-year kidney graft survival after KTA and SPKT, although 3-year glomerular filtration rate (GFR) was higher in the KTA group [12]. Gutierrez et al. reported lower rates of delayed graft function and better early renal function after SPKT compared with cadaveric KTA, without a significant increase in renal surgical complications [17].

Notably, these registry analyses counted events from the date of transplant and retained early post-operative deaths, mitigating survivor-selection bias.

Registry analyses provided a more nuanced perspective. In their propensity-adjusted primary analyses, the two most recent registry studies did not confirm a kidney-graft advantage for SPKT: after matching, Catarinella et al. reported comparable kidney graft failure between SPKT and KTA (HR 0.99, 95% CI 0.94–1.04), and after overlap weighting Budhiraja et al. found no significant difference in kidney graft survival between SPKT and deceased-donor kidney transplantation [24,25].

Young et al. and Barlow et al. reported that LDKT may achieve equivalent or superior kidney graft outcomes compared with SPKT, whereas SPKT appeared to perform more favorably when compared with DDKT [18,21]. Overall, the available evidence suggests that kidney graft outcomes after SPKT are influenced by donor source and comparator type, with the greatest relative benefit observed in comparisons with deceased-donor KTA.

### 3.5. Cardiovascular Outcomes

Evidence regarding cardiovascular outcomes was limited but clinically relevant. Biesenbach et al. reported lower HbA1c levels, lower triglyceride concentrations and a reduced long-term macrovascular disease burden after SPKT compared with KTA following approximately 10 years of follow-up [22]. However, interpretation is limited by the small sample size and the survivor-enriched design, which included only recipients with graft function beyond 5 years.

Lange et al. reported fewer post-transplant cardiovascular events after SPKT than after KTA in a propensity-matched cohort and also observed more favorable cardiovascular and metabolic risk profiles among SPKT recipients [23].

In the unmatched comparison, Catarinella et al. also reported a lower incidence of major adverse cardiovascular events among SPKT recipients; however, this difference did not persist after propensity-score matching (HR 0.99, 95% CI 0.94–1.05), indicating that the apparent cardiovascular advantage was largely attributable to baseline differences between recipients rather than to pancreas transplantation itself [24].

Taken together, these findings suggest that sustained pancreas graft function may contribute to improved cardiovascular risk profiles after transplantation. However, the certainty of this evidence remains limited by the observational design of the available studies, potential residual confounding and selection bias.

### 3.6. Metabolic Outcomes and Pancreas Graft Function

The most recognized benefit associated with SPKT consists of improved metabolic control. Studies involving recipients with a functioning pancreas graft uniformly reported insulin independence or near-normal glycemic control, lower HbA1c levels and more favorable metabolic profiles than those observed after KTA. These findings are biologically linked with restoration of endogenous insulin secretion following successful pancreas transplantation.

However, in the long-term, the metabolic advantage depends on sustained pancreas graft function, in fact improved glycemic control may be attenuated by pancreas graft failure or offset by the higher early morbidity associated with the SPKT procedure.

### 3.7. Complications, Morbidity and Surgical Trade-Offs

SPKT is consistently associated with greater procedural complexity than KTA. Historical cohorts reported substantial infectious morbidity, higher rates of surgical complications and a non-negligible risk of pancreatectomy. Although contemporary studies suggest improved perioperative outcomes, pancreas graft loss, acute rejection, readmissions and longer hospitalization remain important considerations.

Overall, the available evidence indicates that SPKT is associated with higher early morbidity than KTA, whereas its potential long-term benefits are largely dependent on successful and durable pancreas graft function.

### 3.8. Quantitative Synthesis of SPKT Versus LDKT Outcomes

Among the included studies, only Young et al. [18] and Barlow et al. [21] provided directly comparable adjusted hazard ratios with corresponding 95% confidence intervals for both patient mortality and kidney graft failure, allowing quantitative synthesis. Young et al. [18] reported significantly lower adjusted risks of kidney graft failure (HR 0.71, 95% CI 0.61–0.83) and patient death (HR 0.78, 95% CI 0.65–0.94) among LDKT recipients compared with SPKT recipients. Barlow et al. [21] similarly reported a lower adjusted risk of kidney graft failure following LDKT (HR 0.60, 95% CI 0.38–0.94), whereas the difference in patient survival did not reach statistical significance (HR 0.71, 95% CI 0.47–1.06).

**Figure 2:**
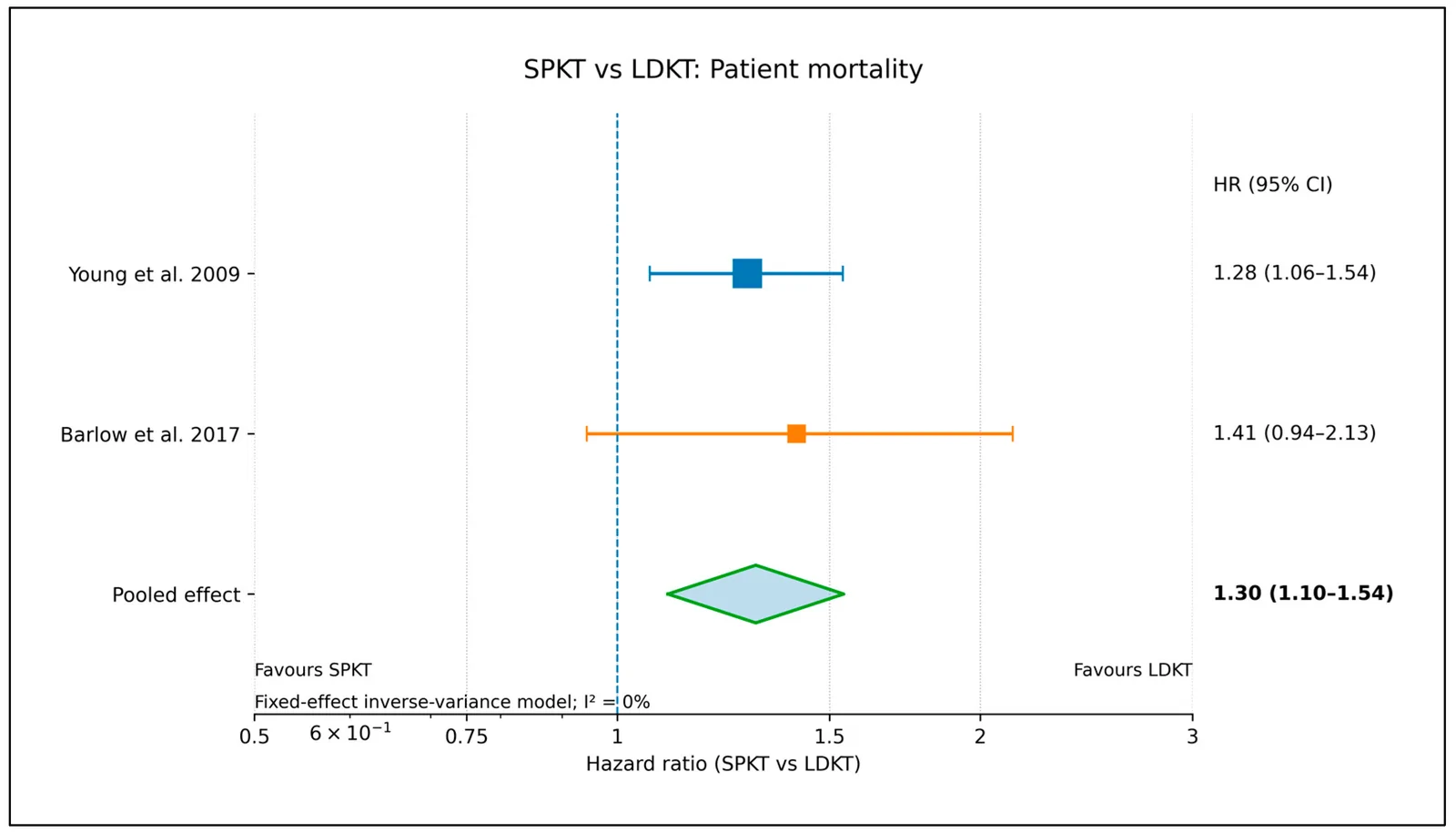
Forest plot of patient mortality comparing simultaneous pancreas–kidney transplantation (SPKT) and living-donor kidney transplantation (LDKT). Hazard ratios were standardized to represent SPKT versus LDKT through reciprocal transformation of studies originally reporting outcomes as LDKT versus SPKT. Values greater than 1.0 indicate a higher mortality risk associated with SPKT, whereas values lower than 1.0 favor SPKT. The pooled estimate was calculated using an inverse-variance fixed-effect model [18,21].

**Figure 3:**
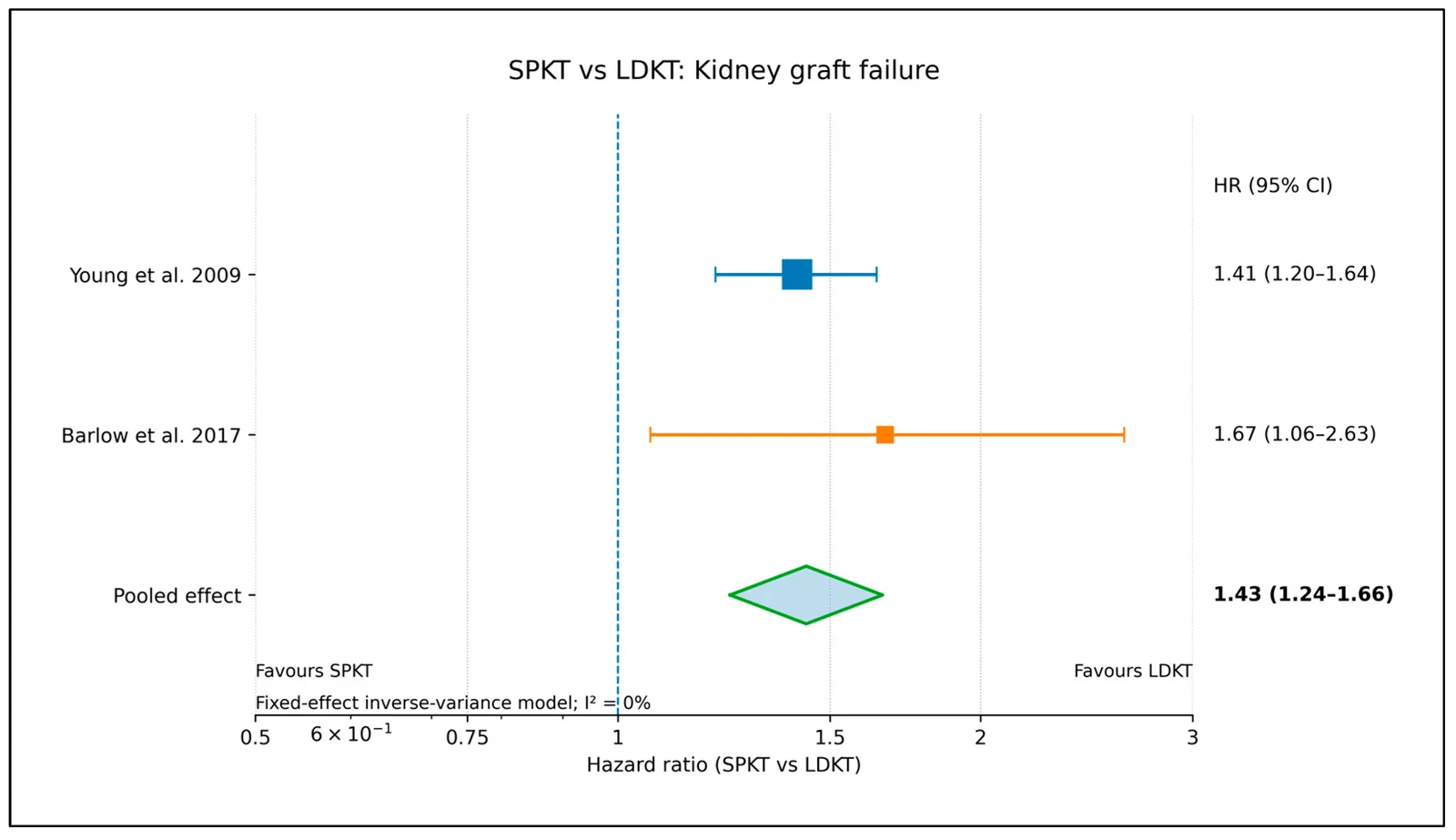
Forest plot of kidney graft failure comparing simultaneous pancreas–kidney transplantation (SPKT) and living-donor kidney transplantation (LDKT). Hazard ratios were standardized to represent SPKT versus LDKT through reciprocal transformation of studies originally reporting outcomes as LDKT versus SPKT. Values greater than 1.0 indicate a higher risk of kidney graft failure associated with SPKT, whereas values lower than 1.0 favor SPKT. The pooled estimate was calculated using an inverse-variance fixed-effect model [18,21].

After harmonization of effect direction to represent SPKT versus LDKT, pooled analysis demonstrated that SPKT was associated with a significantly higher risk of patient mortality compared with LDKT (HR 1.30, 95% CI 1.10–1.54; I^2^ = 0%) (Figure 2). Similarly, SPKT was associated with a significantly higher risk of kidney graft failure (HR 1.43, 95% CI 1.24–1.66; I^2^ = 0%) (Figure 3).

Although several additional studies compared SPKT and DDKT, quantitative synthesis was not feasible because of substantial heterogeneity in statistical reporting, outcome definitions, and follow-up duration. Consequently, these studies were analyzed narratively rather than quantitatively pooled.

I^2^ was 0%, which indicates no statistically detectable heterogeneity; however, with only two observational registry studies and differences in populations and analytic approaches, true clinical heterogeneity cannot be excluded. Overall, the results indicate that, when a suitable living donor is available, LDKT may provide superior patient survival and kidney graft outcomes compared with SPKT. However, they should be interpreted alongside the well-established metabolic and endocrine benefits of SPKT, including insulin independence and long-term glycemic control.

## 4. Discussion

This systematic review suggests that SPKT provides a consistent metabolic advantage over KTA in adults with T1DM and ESRD, whereas its survival and kidney-graft benefits appear to be dependent on comparator type and patient selection. The most favorable comparative results were generally observed when SPKT was compared with deceased-donor or mixed KTA cohorts [15,16,19,23]. In their propensity-adjusted primary analyses, however, the two most recent and largest registry studies did not confirm this pattern, with SPKT showing no significant patient-survival or kidney-graft advantage over deceased-donor KTA after propensity-score matching or weighting [24,25]. In contrast, living-donor kidney transplantation frequently achieved equivalent or superior patient and kidney-graft outcomes while avoiding the additional surgical complexity associated with pancreas transplantation [18,21].

A recurrent finding across the included studies is the central importance of pancreas graft function. Recipients with sustained pancreas graft function consistently experience insulin independence, improved glycemic control and more favorable metabolic profiles. Some studies also suggested potential cardiovascular benefits associated with successful pancreas transplantation [22,23,24]. However, these advantages may be attenuated when pancreas graft failure occurs, particularly during the early post-transplant period.

A related methodological consideration concerns the analytic population of the included studies. Because the survival benefit of SPKT is often framed in terms of ‘successful’ transplantation or of recipients with a functioning pancreas graft, it is important to distinguish analyses performed on an intention-to-treat (as-transplanted) basis from those conditioned on graft success. All registry-based analyses, and in particular the two studies contributing to the quantitative synthesis (Young et al. [18] and Barlow et al. [21]), together with Manske et al. [10], Reddy et al. [16], Sung et al. [19], Catarinella et al. [24] and Budhiraja et al. [25], analyzed all transplanted patients from the time of transplantation and therefore captured early post-transplant mortality and technical or graft failures. In these intention-to-treat analyses the early hazard of SPKT is fully reflected; indeed several of them (Manske et al. [10], Reddy et al. [16] through 18 months, Young et al. [18] and Barlow et al. [21]) show an early or overall survival disadvantage for SPKT relative to the comparator. By contrast, a minority of analyses conditioned on graft function and are therefore vulnerable to survivorship bias: most notably, Biesenbach et al. [22] included only recipients whose grafts survived beyond five years, and several studies additionally reported subgroup analyses restricted to recipients with a functioning pancreas graft (Reddy et al. [16], Barlow et al. [21] and Lange et al. [23]). Statements in this review referring to the benefits of ‘successful’ SPKT, or to outcomes obtained when pancreas graft function was maintained, derive from these conditional analyses and should be interpreted with caution, because they exclude patients who died or lost graft function early after transplantation. Reassuringly, the contemporary analyses that retained all transplanted patients and adjusted rigorously for selection (Catarinella et al. [24] and Budhiraja et al. [25]) found no residual survival or graft advantage for SPKT, indicating that earlier ‘successful-SPKT’ comparisons are likely to have overestimated the benefit of pancreas transplantation.

The exploratory quantitative synthesis performed in the present review provides additional support for the comparative findings observed across the included studies. By pooling the only two studies reporting directly comparable adjusted hazard ratios, SPKT was associated with a higher risk of both patient mortality and kidney graft failure compared with LDKT. These findings are consistent with the individual observations of Young et al. [18] and Barlow et al. [21], both of which favored LDKT for kidney graft outcomes and suggested either comparable or improved patient survival relative to SPKT. Importantly, these results should not be interpreted as evidence that LDKT is universally superior to SPKT. Rather, they likely reflect the combined influence of shorter waiting times, reduced dialysis exposure, lower perioperative risk, and the elective nature of living-donor transplantation. Conversely, the quantitative synthesis does not capture several important advantages of SPKT, including sustained insulin independence, improved glycemic control, and the potential long-term attenuation of diabetes-related complications. Therefore, the pooled estimates should be interpreted within the broader clinical context and viewed as complementary to, rather than replacing, individualized patient-centered decision making.

The findings of this review support an individualized approach to treatment selection. SPKT may be particularly attractive in suitable candidates without access to a living donor and in patients for whom long-term metabolic control and insulin independence are major therapeutic goals. Conversely, LDKT remains an excellent option when a high-quality living donor is available, especially when transplantation can be performed pre-emptively or after limited dialysis exposure.

Consideration should also be given to studies reflecting an older surgical and immunosuppressive era, preventing generalization directly to contemporary practice. Practical considerations related to organ availability also bear on treatment selection. Because deceased-donor pancreas grafts are considerably scarcer than kidney grafts, waiting times for SPKT are generally longer than for KTA, and the additional time on dialysis exposes candidates to continued progression of diabetic micro- and macrovascular complications while they wait. This trade-off—between a potentially longer wait for a dual-organ transplant and earlier isolated kidney transplantation—is central to individual decision-making, yet robust data on recipients’ own preferences between SPKT and KTA are scarce. To our knowledge, few studies have formally assessed patient-reported preferences or decisional priorities in this setting, which represents an important evidence gap; structured shared decision-making and dedicated research into recipient preferences are therefore warranted to complement the survival and metabolic outcomes summarized here.

The comparator against which SPKT is judged is itself strongly influenced by health-system context. The availability of living-donor kidney transplantation varies markedly between countries and programmes—being comparatively limited in some systems and highly developed in others (for example, the contrast between living-donation activity in Spain, UK, The Netherlands and in the Scandinavian countries)—so that the SPKT versus LDKT comparison is pertinent only where a suitable living donor is genuinely available. For the substantial proportion of candidates without a living donor, the real-world choice lies between SPKT and deceased-donor KTA. This distinction matters because the evidence is comparator-dependent: whereas LDKT frequently matched or outperformed SPKT, the comparison of SPKT with deceased-donor KTA was generally more favorable to SPKT in unadjusted analyses—although, as noted above, this advantage was substantially attenuated in the most rigorously adjusted contemporary cohorts. The applicability of our findings therefore depends on local donor availability, and the promising SPKT-versus-LDKT signal should not be extrapolated to settings in which the practical alternative is deceased-donor KTA.

Recent contemporary evidence further supports the importance of recipient selection when interpreting comparative outcomes after transplantation. In a large propensity score–matched international cohort, Catarinella et al. demonstrated that the apparent advantages of SPKT in terms of patient survival, kidney graft survival and cardiovascular outcomes were substantially attenuated after adjustment for baseline differences between recipients [24]. Nevertheless, SPKT recipients maintained significantly superior long-term glycemic control, with persistently lower HbA1c levels throughout follow-up, supporting the concept that the principal benefit of successful pancreas transplantation may reside in durable metabolic restoration and insulin independence rather than in a universal survival advantage [34]. Importantly, sensitivity analyses restricted to recipients with type 1 diabetes yielded findings consistent with the primary analysis, reinforcing the robustness of these observations and suggesting that the metabolic advantages of SPKT may remain clinically meaningful even when survival differences are less pronounced [24].

More broadly, the included studies span three decades (1995–2025), and both the intervention and its alternatives changed substantially over this period. Surgical technique, organ preservation, induction and maintenance immunosuppression and perioperative care have all improved, so that the high early mortality reported in the earliest SPKT cohorts is unlikely to reflect contemporary results; combining studies from the 1990s with those from the 2000s and 2020s therefore introduces important temporal heterogeneity. In parallel, the medical management of type 1 diabetes has advanced considerably, with continuous glucose monitoring and automated (closed-loop) insulin-delivery systems now achieving degrees of glycemic control that were previously attainable only through pancreas transplantation. For a well-controlled candidate using such technologies, proceeding earlier to kidney transplantation alone—particularly from a living donor—may be a reasonable alternative to a longer wait for SPKT, further reinforcing the need for an individualized, era-aware interpretation of the comparative evidence.

Strengths: Strengths of this review include the use of a predefined PICO framework, a systematic PubMed/MEDLINE search strategy, explicit eligibility criteria, structured full-text assessment, separation of core and supportive evidence and a transparent narrative synthesis tailored to the heterogeneity of the available literature.

Limitations: All included studies were observational, making the evidence susceptible to residual confounding and selection bias. Considerable heterogeneity was present with respect to donor source, follow-up duration, outcome definitions and analytical methods. In addition, some studies originated from overlapping registry datasets and transplant practices evolved substantially over the study period, potentially limiting the comparability of historical and contemporary cohorts. Consequently, the findings should be interpreted cautiously and supportive of clinical decision-making rather than definitive evidence of superiority for either transplant strategy.

Limitations of the quantitative synthesis warrant emphasis. The meta-analysis pooled only two studies (Young et al. [18] and Barlow et al. [21]), the sole included reports providing directly comparable adjusted hazard ratios for both patient mortality and kidney graft failure in the SPKT versus LDKT comparison. Combining only two estimates provides limited statistical power and, more importantly, does not permit any reliable assessment of between-study heterogeneity or of publication and small-study bias; the observed I^2^ of 0% is therefore statistically uninformative rather than reassuring. Accordingly, the pooled estimates carry a correspondingly high risk of bias and should be regarded as exploratory and hypothesis-generating rather than confirmatory.

A further consideration is the degree of patient overlap between data sources. The two studies combined in the quantitative synthesis are drawn from independent national registries—the United States OPTN/UNOS dataset (Young et al.) [18] and the United Kingdom Transplant Registry (Barlow et al. [21])—and therefore do not share patients, which limits overlap bias for the pooled estimate specifically. Across the wider narrative synthesis, however, several analyses were derived from overlapping United States registries (UNOS/OPTN and the SRTR: Reddy et al. [16], Young et al. [18], Sung et al. [19] and Budhiraja et al. [25]), which draw on the same national transplant population over overlapping eras. These studies are consequently not statistically independent; we did not pool them, and their broadly concordant direction should be interpreted in light of this shared provenance rather than as replication across distinct populations.

## 5. Conclusions

In adults with T1DM and ESRD, SPKT with sustained pancreas graft function provides durable metabolic benefits and may improve long-term outcomes compared with deceased-donor or mixed KTA in selected recipients. However, current evidence does not support a consistent survival or kidney-graft advantage of SPKT over living-donor KTA. Treatment decisions should therefore be individualized according to living donor availability, dialysis exposure, cardiovascular risk, surgical risk and the likelihood of durable pancreas graft function. The value of SPKT lies not simply in replacing renal function but in restoring metabolic physiology.

## Figures and Tables

**Table 1:** PICO-based inclusion and exclusion criteria for study selection.

Domain	Inclusion Criteria	Exclusion Criteria
Population	Adults with T1DM and ESRD or advanced CKD undergoing kidney transplantation	Pediatric populations; type 2 or mixed diabetes populations without separable T1DM data; pregnancy/gestational diabetes populations
Intervention	SPKT transplantation	Pancreas-alone transplantation or pancreas-after-kidney transplantation without extractable SPKT vs KTA comparison
Comparator	KTA, including living-donor, deceased-donor/cadaveric or mixed-donor cohorts	No KTA comparator; comparator limited to non-diabetic kidney recipients
Outcomes	Patient survival, kidney graft survival/function, pancreas graft function, cardiovascular outcomes, metabolic control, complications, quality of life or other clinically relevant outcomes	No original extractable clinical outcome data relevant to the PICO
Study type	Original clinical comparative studies in English	Narrative reviews, systematic reviews, editorials, letters without original data, case reports, animal studies, technical duplicates

**Table 2:** Characteristics of the included core comparative studies.

Ref.	Study	Country	Study Design	Comparator	Sample Size (SPKT/KTA)	Follow-Up	Main Outcome
[10]	Manske et al. 1995	USA	Single-centre cohort	Cadaveric and living-donor KTA	54/111	3 years	Patient survival
[11]	Cattral et al. 1998	Canada	Retrospective cohort	Living-donor and cadaveric KTA	20/39	Short-term	Patient and kidney graft survival
[12]	Douzdjian et al. 1998	USA	Retrospective cohort	KTA	42/60	Up to 5 years	Kidney graft survival and function
[13]	Rayhill et al. 2000	USA	Longitudinal cohort	Living-donor and cadaveric KTA	379/426	Up to 5 years	Patient and graft survival
[14]	Navarro et al. 2001/2002	Spain	Retrospective cohort	Cadaveric KTA	29/15	2 years	Patient and kidney graft survival
[15]	Mohan et al. 2003	Ireland	Comparative cohort	KTA	50/51	8 years	Patient survival
[16]	Reddy et al. 2003	USA	UNOS registry cohort	Living-donor and cadaveric KTA	Registry-based	8 years	Long-term survival
[17]	Gutierrez et al. 2007	Spain	Matched comparative cohort	Cadaveric KTA	20/25	6 months	Early graft function and morbidity
[18]	Young et al. 2009	USA	OPTN/UNOS registry cohort	LDKT and DDKT	5352/6010	72 months	Patient and kidney graft survival
[19]	Sung et al. 2015	USA	SRTR registry cohort	KTA	7308/4653	10 years	Adjusted survival outcomes
[20]	Dinckan et al. 2015	Turkey	Retrospective cohort	KTA	21/30	5 years	Kidney graft survival
[21]	Barlow et al. 2017	United Kingdom	Registry cohort	LDKT	1739/385	Median 3–4 years	Patient and kidney graft survival
[22]	Biesenbach et al. 2005	Austria	Long-term cohort	KTA	12/10	~10 years	Cardiovascular and metabolic outcomes
[23]	Lange et al. 2021	Germany	Propensity-matched cohort	KTA	42/21	Mean 7.7 years	Survival and cardiovascular outcomes
[24]	Catarinella et al. 2025	International/global cohort	Retrospective propensity score–matched cohort	KTA, mainly deceased-donor KTA	3679/27,062	Median ~6 years	Patient survival, graft survival, cardiovascular outcomes and metabolic control
[25]	Budhiraja et al. 2025	USA	Registry-based cohort with overlap propensity weighting	DDKT	5752/16,793	Median 46 months for patient survival; 36 months for graft survival	Patient survival, kidney graft survival, acute rejection and hospital readmission

**Table 3:** Risk of bias assessment (Newcastle–Ottawa-style scoring).

Study	Selection/4	Comparability/2	Outcome/3	Total/9	Risk-of-Bias Judgement
Manske 1995 [10]	3	1	2	6	Moderate/high
Cattral 1998 [11]	3	1	1	5	Moderate/high
Douzdjian 1998 [12]	3	1	2	6	Moderate
Rayhill 2000 [13]	3	1	2	6	Moderate
Navarro 2002 [14]	2	1	2	5	Moderate/high
Mohan 2003 [15]	3	1	2	6	Moderate
Reddy 2003 [16]	4	2	2	8	Low/moderate
Gutiérrez 2007 [17]	3	1	2	6	Moderate
Young 2009 [18]	4	2	2	8	Low/moderate
Sung 2015 [19]	4	2	3	9	Low
Dinckan 2015 [20]	2	1	2	5	Moderate/high
Barlow 2017 [21]	4	2	2	8	Low/moderate
Biesenbach 2005 [22]	2	1	2	5	Moderate/high
Lange 2021 [23]	3	2	2	7	Low/moderate
Catarinella 2025 [24]	4	2	2	8	Low/moderate
Budhiraja 2025 [25]	4	2	3	9	Low

## Data Availability

The original contributions presented in this study are included in the article/supplementary material. Further inquiries can be directed to the corresponding author(s).

## Supplementary Materials

The following supporting information can be downloaded at: https://www.mdpi.com/article/10.3390/medsci14040454/s1, Table S1. Detailed characteristics, principal findings and interpretive assessment of the included core comparative studies. Table S2. Secondary/supportive studies included for contextual interpretation. Supplementary Table S3. Full-text studies assessed for eligibility but not retained in the final evidence synthesis.
