# Supplementary material for: Simultaneous Pancreas-Kidney Transplantation Versus Kidney Transplantation Alone in Type 1 Diabetes: Does Pancreas Transplantation Improve Clinical Outcomes? A Systematic Review and Exploratory Meta-Analysis
Source: Med Sci (Basel). 2026 Aug 3;14(4):454. doi: 10.3390/medsci14040454 (PMC13515573; doi:10.3390/medsci14040454)
Supplement: Supplementary file 1 [file medsci-14-00454-s001.zip › medsci-4447334-supplementary.pdf]

Supplementary Table S1. Detailed characteristics, principal findings and interpretive assessment of the included core comparative studies

Study	Year	Country	Design / data source	Population	SPKT	KTA / comparator	Follow-up	Main verified result	Interpretive note
Manske et al. [10]	1995	USA	Non-randomized single-centre cohort	IDDM/T1DM renal transplant candidates	54 KP/SPKT	46 cadaveric KTA; 65 living-related KTA; 8 PAK	3 years	3-year patient survival was lower after KP/SPKT than cadaveric KTA (68% vs 90%; p=0.01). Excess early mortality was mainly infection-related; 14/54 KP/SPKT recipients required pancreatectomy.	Historical era; important early safety signal but limited applicability to contemporary practice.
Cattral et al. [11]	1998	Canada	Retrospective single-centre cohort	T1DM with end-stage diabetic nephropathy	20 SPK	20 living-related KTA; 19 cadaveric KTA	Minimum 4 months; mean follow-up 11 +/- 6 mo (SPK), 29 +/- 16 mo (KTA-LR), 24 +/- 16 mo (KTA-CAD)	Patient survival: 95% SPK, 100% KTA-LR, 84% KTA-CAD (NS). Kidney graft survival: 95%, 95%, 84% (NS). Pancreas graft survival: 90%. Functioning pancreas grafts achieved normoglycaemia.	Small, short-term cohort; underpowered for survival.
Douzdjian et al. [12]	1998	USA	Retrospective single-centre cohort	Type 1 diabetic kidney or kidney-pancreas transplant recipients, 1990-1996	42 SPK	60 KTA (42 cadaveric; 18 living-related)	Function to 3 years; survival to 5 years	Kidney graft survival similar at 1 and 5 years: KTA 87%/44% vs SPK 89%/47% (p=0.8). GFR at 3 years higher after KTA (60 +/- 22 vs 42 +/- 16 ml/min; p=0.03).	Small cohort; graft-function detail, no survival advantage for SPK.
Rayhill et al. [13]	2000	USA	Longitudinal single-centre database	Diabetic ESRD recipients, 1986-1996	379 SPK	43 HLA-identical LRD; 87 haplotype-identical LRD; 296 cadaveric KTA	Up to 5 years	Patient and graft survival were similar for SPK and living-related donor groups, but significantly lower after cadaveric KTA. Delayed graft function was more common after cadaveric KTA.	Comparator-stratified cohort; living donor KTA remained comparable to SPK.
Navarro et al. [14]	2002	Spain	Retrospective single-centre cohort	T1DM with ESRD receiving cadaveric kidney or SPK transplantation	29 SPK	15 cadaveric KTA	2 years	2-year kidney graft survival: 81% SPK vs 73% KTA (p<0.05). Patient survival: 92% SPK vs 86% KTA (p=0.07). Pancreas graft survival: 75%. Rejection was numerically lower in SPK (20.6% vs 40%; NS).	Small historical cadaveric-donor cohort.

Study	Year	Country	Design / data source	Population	SPKT	KTA / comparator	Follow-up	Main verified result	Interpretive note
Mohan et al. [15]	2003	Ireland	Retrospective comparative cohort	T1DM with ESRD transplanted 1992-2002	50 SPK	51 KTA	Up to 8 years	Patient survival at 1/3/5/8 years was 96/93/89/77% after SPK and 93/75/57/47% after KTA (p=0.018 at 8 years).	Single-centre cohort with coronary screening; observational.
Reddy et al. [16]	2003	USA	UNOS registry cohort	T1DM renal transplant recipients, 1987-1996	4,602 SKPT	3,991 living-donor KTA; 9,956 cadaveric KTA	8 years	8-year survival: 72% SKPT, 72% living-donor KTA, 55% cadaveric KTA. SKPT had higher mortality than living-donor KTA during first 18 months (HR 2.2; p<0.001) but lower risk thereafter (RR 0.86; p<0.02).	Large registry; comparator-dependent and era-sensitive.
Gutierrez et al. [17]	2007	Spain	Matched comparative cohort	Selected T1DM patients with ESRD receiving SPKT or cadaveric KTA	20 SPKT	25 cadaveric KTA	6 months	Delayed graft function was less frequent after SPKT (5% vs 32%; p<0.05). Creatinine was lower at discharge and 6 months after SPKT. No significant increase in renal surgical complications.	Short-term renal function/morbidity study.
Young et al. [18]	2009	USA	OPTN/UNOS registry cohort	Adult T1DM recipients aged 20-59 receiving first kidney transplant, 2000-2007	5,352 SPKT	3,309 LDKT; 2,701 DDKT	72 months	LDKT had lower adjusted risk of kidney graft failure (HR 0.71; 95% CI 0.61-0.83) and patient death (HR 0.78; 95% CI 0.65-0.94) versus SPKT. SPKT had better unadjusted outcomes than DDKT, partly due to donor/recipient factors.	High-value registry evidence; eligible for SPKT vs LDKT HR meta-analysis.
Sung et al. [19]	2015	USA	SRTR registry cohort with adjusted survival-curve methods	Adult T1DM recipients receiving SPK or KTA, 1998-2009	7,308 SPK	4,653 KTA	10 years	Adjusted restricted mean kidney graft survival time was 0.18 years greater for SPK (p=0.045), and patient survival time was 0.17 years greater (p=0.033). Authors considered the advantage statistically significant but clinically small.	Large registry; avoids simple HRs because hazards were non-proportional.
Dinckan et al. [20]	2015	Turkey	Retrospective single-centre cohort	Type 1 diabetes-related ESRD transplant cases, 2003-2010	21 SPK	30 living-donor KA; 34 PAK third arm	5 years	5-year kidney graft survival: KA 86.7%, SPK 95.2%, PAK 97.1%. One death in KA and one in SPK.	Small single-centre cohort; includes PAK arm.

Study	Year	Country	Design / data source	Population	SPKT	KTA / comparator	Follow-up	Main verified result	Interpretive note
Barlow et al. [21]	2017	United Kingdom	UK Transplant Registry cohort	T1DM with ESRD receiving SPK or LDK, 2001-2014	1,739 SPK	385 LDK	Median 3.3 years (SPK) and 3.9 years (LDK)	No univariate difference in patient survival (p=0.435) or kidney graft survival (p=0.204). LDK had lower kidney graft failure risk (HR 0.60; 95% CI 0.38-0.94); patient survival was not significantly different (HR 0.71; 95% CI 0.47-1.06). Functioning pancreas graft associated with better outcomes.	High-value registry evidence; eligible for SPKT vs LDKT HR meta-analysis.
Biesenbach et al. [22]	2005	Austria	Retrospective long-term survivor-enriched cohort	T1DM recipients with functioning grafts >5 years	12 SPKT	10 KTA	Mean 124 vs 122 months	HbA1c and triglycerides were lower after SPKT. At approximately 10 years, macrovascular disease burden was lower after SPKT, limited by small sample size.	Cardiovascular/metabolic study; survivor-enriched and underpowered.
Lange et al. [23]	2021	Germany	Retrospective cohort with 2:1 propensity-score matching	IDDM/T1DM with ESRD, 1998-2019	42 SPKT after PSM (100 initial)	21 KTA after PSM (27 initial)	Mean 7.7 +/- 3.95 years	After PSM, mortality was lower after SPKT (HR 0.63; 95% CI 0.49-0.89). Post-transplant cardiovascular events were lower after SPKT (7% vs 28%; p=0.02). HbA1c, blood pressure and LDL/HDL ratio improved after SPKT.	Contemporary single-centre PSM study; small matched sample.
Catarinella et al. [24]	2025	International / global cohort	Retrospective TriNetX cohort with 1:1 propensity-score matching	Diabetes and ESRD recipients aged 18-59, 2010-2024; sensitivity analyses for T1D	3,679 SPKT	27,062 KTA before matching	Up to 10 years; median approx. 6 years	After matching, survival HR 1.00 (0.90-1.10), kidney graft failure HR 0.99 (0.94-1.04) and cardiovascular events HR 0.99 (0.94-1.05) no longer differed. HbA1c remained lower after SPKT (6.2% vs 6.6%; p<0.001).	Contemporary real-world evidence; mixed diabetes population, sensitivity analyses support robustness.
Budhiraja et al. [25]	2025	USA	SRTR registry cohort with overlap propensity weighting	Diabetic recipients of SPKT or DDKT, 2014-2023; subgroup analyses for T1D/BMI	5,752 SPKT	16,793 DDKT	Median 46 months for patient survival; 36 months for graft survival	After overlap weighting, no significant difference in patient mortality (DDKT vs SPKT HR 0.85; 0.64-1.13) or kidney graft failure (HR 0.86; 0.66-1.11). SPKT had higher 1-year treated rejection (OR 2.80; 1.75-4.49) and readmission (OR 2.05; 1.62-2.60).	Contemporary registry evidence; mixed diabetes population and DDKT-only comparator; main message is no adjusted survival/graft advantage, with higher early morbidity.

Abbreviations: DDKT, deceased-donor kidney transplantation; ESRD, end-stage renal disease; GFR, glomerular filtration rate; HR, hazard ratio; IDDM, insulin-dependent diabetes mellitus; KA/KTA, kidney-alone transplantation/kidney transplantation alone; LDK/LDKT, living-donor kidney transplantation; LRD, living-related donor; NS, not significant; PAK, pancreas-after-kidney transplantation; PSM, propensity-score matching; SPK/SPKT, simultaneous pancreas-kidney transplantation; T1D/T1DM, type 1 diabetes mellitus.

Supplementary Table S2. Secondary/supportive studies included for contextual interpretation

Study	Year	Country	Design/category	Population/comparator	Reason for supportive inclusion	Main verified finding	Use in review
Tyden et al. [26]	1997	Sweden	Prospective 10-year follow-up report	IDDM patients after combined pancreas-kidney transplantation compared with kidney transplantation alone/failed pancreas control group; 14 combined pancreas-kidney recipients and 15 kidney-alone controls.	Supportive long-term metabolic, neurologic, quality-of-life and survival evidence; small cohort and likely overlapping Swedish experience, therefore not treated as core comparative evidence.	Pancreas-kidney recipients maintained long-term normoglycemia, had improved neuropathy/autonomic function and better quality-of-life scores; 10-year patient survival was reported as 79% versus 20% in kidney-only controls.	Contextual support for the potential long-term metabolic and quality-of-life benefit of durable pancreas graft function.
Nikkel et al. [27]	2013	USA	USRDS registry cohort; secondary outcome study	Adults with T1DM undergoing SPKT or kidney-alone transplantation between 2000 and 2006; 4,933 SPKT and 6,212 KTA recipients.	Relevant secondary endpoint outside the primary review focus; evaluates fracture risk rather than patient survival, kidney graft survival, cardiovascular outcomes or metabolic control.	Hospitalization-associated fractures were less frequent after SPKT than KTA (4.7% vs 5.9%). After adjustment, SPKT was associated with reduced overall fracture risk, particularly in men (HR about 0.69; propensity-adjusted HR about 0.67). No comparable reduction was observed in women.	Supportive evidence that restoration of endocrine/metabolic physiology may have clinically relevant effects beyond survival and graft endpoints, but not part of the primary comparative synthesis.
Venkatanarasimhamoorthy and Barlow [21]	2018	United Kingdom	Narrative review	Review of evidence comparing SPKT and LDKT in patients with T1DM and advanced CKD/ESRD.	No original clinical data; retained only for contextual interpretation of decision-making between SPKT and LDKT.	Concluded that SPKT versus LDKT choice should not be based only on patient or graft survival. It emphasized individual circumstances, comorbidities, waiting time, dialysis exposure, patient preference and the importance of sustained pancreas graft function.	Contextual support for the discussion of individualized transplant selection and procedure-related trade-offs.

Abbreviations: CKD, chronic kidney disease; ESRD, end-stage renal disease; HR, hazard ratio; IDDM, insulin-dependent diabetes mellitus; KTA, kidney transplantation alone; LDKT, living-donor kidney transplantation; SPKT, simultaneous pancreas-kidney transplantation; T1DM, type 1 diabetes mellitus; USRDS, United States Renal Data System.

Supplementary Table S3. Full-text studies assessed for eligibility but not retained in the final evidence synthesis

Study	Year	Category of non-inclusion	Reason for exclusion
Pérez et al. [28]	2001	Overlapping cohort	Superseded by an updated Pérez/Navarro cohort with more complete outcome data; the later/more complete dataset was retained to avoid double counting.
Tibell et al. [29]	1997	Survivor-enriched selected cohort	Included only recipients with graft function beyond 2 years, creating substantial survivor-selection bias; not retained in the final core synthesis.
Biesenbach et al. [30]	2000	Overlapping/earlier cohort	Earlier and smaller report on macrovascular outcomes, superseded by the later 2005 Biesenbach study with longer follow-up and more complete cardiovascular/metabolic assessment.
Hörstrup et al. [31]	2005	Wrong comparator population	Compared SPKT recipients with non-diabetic cadaveric kidney transplant recipients rather than diabetic KTA recipients.
Donigan et al. [32]	2004	No relevant post-transplant outcome	Evaluated pre-transplant cardiovascular risk factors and baseline characteristics rather than post-transplant comparative clinical outcomes.
Montiel-Casado et al. [33]	2013	Wrong comparator/intervention	Compared SPK with pancreas-after-kidney and pancreas-alone transplantation without a kidney-transplant-alone comparator relevant to the review question.
